# Love and Realism

**DOI:** 10.1007/s10699-015-9471-6

**Published:** 2015-10-17

**Authors:** Pieter Lemmens

**Affiliations:** Nijmegen, Netherlands

**Keywords:** Digital technologies, Technical condition, General intellect, Post-phenomenology, Dialectics, Politics of technology

## Abstract

In this reply I try to show that, contrary to Milberry’s apparent assertion, the general intellect of the multitude does not have the explanatory robustness she accredits to it (following both Virno and the Hardt and Negri of the *Empire* trilogy). Digital network technologies are currently overwhelmingly effective in proletarianizing and disempowering the cognitariat and only an active technopolitics of deproletarianization could reverse this hegemonic situation. In my response to Verbeek, I attempt to correct his misinterpretation (shared by Milberry) of the Stieglerian approach as being dialectical in nature and show that, far from reinstating the humanist dichotomy between human beings and technologies, my analysis assumes their *original*, albeit fundamentally ambiguous and even ‘uncanny’ [*unheimlich*] interconnection. I conclude with pointing out some implications of this view for a ‘really realistic’ political theory of technology.

First of all I want to thank both commentators for taking the time and the effort to write their thoughtful and carefully composed responses to my article. Both are very much appreciated.

I’ll start off my reply with Milberry’s response, which seems most sympathetic to the overall argument and general philosophical and political approach of the article, and mostly offers affirmative comments, yet also presents some critical remarks. Before that, however, I feel compelled to correct two apparent misunderstandings. First, although I largely approve of Feenberg’s critical theory of technology, my notions of ambivalence and creative appropriation are adapted from Stiegler and not taken from Feenberg, and are as such based on the former’s pharmacological conception of technology, which certainly has some affinities with Feenberg’s ambivalence theory but, as I write in the article, is rooted in an elaborate ‘onto-anthropological’ and ‘onto-anthropogenetic’ understanding of the human life form as a *technical* life form, one that is lacking in Feenberg and that fully explains *why* and *how* technologies are fundamentally and irreducibly ambivalent and in constant need of creative (re)appropriation (or ‘adoption’ in Stiegler’s terminology). Second, the pharmacological approach to technology and technological change is decidely not dialectical, as Milberry repeatedly yet erroneously infers. But I’ll come back to this in my reply to Verbeek, who shares this misunderstanding.

Referring to both Virno and Hardt and Negri, Milberry argues that the concept of the general intellect, indeed central to my article, may have more explanatory robustness than I acknowledge. Presuming this to mean that it *crucially* explains the growing revolutionary potential of the multitude under current circumstances of immaterial labor, I must reiterate that I find this unconvincing, both empirically and conceptually, at least when this is taken in the sense that it ‘automatically’, as it were, grants labor more autonomy and thus yields it more powerful and assertive vis-à-vis capital. The proletarianizing efficacy of the digital network technologies (DNTs) underpinning the ‘communism of capital’ currently far outweighs their empowering functionality, as a vast and growing body of empirically based literature attests (to mention just a few: Carr 2010, 2015; Crary 2013; Keen 2015; McChesney 2013; Pariser 2011; Rouvroy and Berns 2013) and only a (bottom-up) technopolitics of deproletarianization oriented towards a positive pharmacology—which entails more than simply ‘commonization’—could bring about a turn in this hegemonic tendency.

Of course, this is not the whole story and in particular Berardi’s diagnosis is fairly reductionist, I readily admit, yet it adequately describes certain dominant trends in the working conditions of today’s cognitariat, resonating for instance with Jonathan Crary’s descriptions of 24/7 capitalism (Crary 2013) and the analyses of communicative capitalism’s proletarianizing capture of the libidinal energies of workers and consumers via the digital networks by Jodi Dean, who emphasizes the depoliticizing character of those networks, as they systematically distract subjects from engagement in real political action by soliciting them continuously to online *pseudo*-activity (Dean 2010). As a matter of fact, for Dean, as an active participant in it, Occupy’s success (like that of the other revolts of 2011) should not so much be explained from the political blessings of new social media, but from ‘the physical amassing of people outside’ (Dean 2012, 216). Even Hardt and Negri most recently have recognized the largely disempowering effects of digital media on the thoroughly ‘mediated subjects’ of contemporary capitalism, constantly absorbed as they are in attentional cycles fragmenting their psyches and eroding their (in particular political) affects. Echoing Dean, they write: ‘Facebook, Twitter, the Internet and other kinds of communications mechanisms are useful, but nothing can replace the being together of bodies and the corporeal communication that is the basis of collective political intelligence and action’ (Hardt and Negri 2012, 18).

As for the call for democratic control of technology, which Milberry proposes as a student of Feenberg: as I’ve tried to show in a recent article co-written with Mithun Bantwal-Rao, Joost Jongerden and Guido Ruivenkamp, under capitalist conditions, the call for democratization of technological innovation seems idle since it ‘remains severely limited for the simple reason that there is no incentive for industry to engage in any significant democratization for any reason beyond that of the market […] or public institutional force’ (Bantwal-Rao et al. 2015). Such a call only makes sense if combined with a radical critique of the capitalist order, something that is conspicuously absent in Feenberg’s later, watered-down (from too much wrong-headed concessions to STS, that is) version of critical theory.

Milberry concludes her response with a remarkably idealist critique of my proposal for a pharmacological (re)appropriation and redesign of our digital milieu, arguing that this should be preceded by a change in our ‘social values and mores’ towards more ‘love’, thereby apparently disregarding the ruining effects on self-love and the affective capacities of individuals in general of the omnipresent apparatuses of libidinal capture and exploitation that hegemonically constitute the current ‘sociotechnical foundation of contemporary life’ under capitalist conditions (Stiegler 2009; Berardi 2009). I very much agree that love, also in its social form of *philia* (Aristotle), is key to a better society, but before preaching love, we should—as good Marxists—first consider its current means and conditions of production (or better: destruction) and then think of strategies to transform these.

I will now turn to Verbeek’s response, that is more critical about my general approach, which is implicitly accused of being unrealistic (i.e., in its plea for a ‘realistic political theory of technology’) for basically two reasons: (1) it is, apparently, not empirically informed in that it disregards ‘actual technologies’ and only applies ‘pre-given philosophical frameworks’ ‘from outside’ (that are even suggested to be obsolete) to a technological domain of ‘things themselves’ that are left uninvestigated (just like the traditional, transcendentalist philosophers of technology did); and (2) it remains caught in a dialectic of domination and liberation that is apparently also obsolete and unproductive and should be replaced, or at least complemented, with a hermeneutic approach, present in the allegedly dialectical approach of Stiegler (and obviously also Feenberg) but lacking in my analysis. The ‘realistic’ alternative proposed is a more hermeneutic approach oriented towards ‘the things themselves’, here understood *not* ‘in the phenomenological sense’ (as Verbeek nonetheless claims) but empirically, in the way STS approaches technology—surely neither Husserl nor Heidegger ever thought of ‘*die Sachen selbst*’ as concrete objects; these were precisely ‘bracketed’ in order to attain the proper phenomenological *Sach*-dimension: the acts of transcendental consciousness (like intentionality) for Husserl, modes of being for Heidegger.

Before responding to these critiques, I again feel the need to first correct Verbeek’s (as well as Milberry’s) misinterpretation of Stiegler’s (and my own) approach as being ‘dialectical’. To state it directly: Stiegler’s approach to technology is *not* dialectical. As *organo*logical, it understands the human-technology-society relation with Simondon as *transductive* (and thus not dialectic) in nature and with Nietzsche as traversed by com-posing, rather than op-posing *antagonistic* tendencies; and in this sense Stiegler’s view is close to that of post-autonomist Marxism (Bantwal-Rao et al. 2015). As *pharmaco*logical, it simply *cannot* be dialectical because the *pharmakon*’s ‘negativity’ (its toxicity) can never be ‘sublated’ (*aufgehoben*) as it persists as technical heteronomy and thus calls for an on-going therapy (Stiegler 2015, 129). Explaining this first requires some comments on the notion of the dialectic.

It is my impression that, for Verbeek, this notion only refers to the Marxist struggle of oppression and resistance between labor and capital (as derived from Hegel’s master–slave dialectic). At its origins in Hegel and Marx, however, the dialectic first of all describes the *historical process* of human culture as a process of *exteriorization*, idealistically understood in Hegel as the self-externalization and subsequent internalization of the spirit, materialistically in Marx as the self-production of the human species through its externalized means of production, i.e., technologies or technical organs.

Like Marx, Stiegler also thinks of human evolution and history as based in a process of technical exteriorization (and interiorization). In contrast to Marx, however, who remains an instrumentalist in apprehending technologies as *means* sovereignly determined by the human subject, Stiegler conceives of technologies as *constituting* and *conditioning* the human ‘subject’. Moreover, since technologies—as *pharmaka* compensating for man’s ‘original lack’—are not only curative but *simultaneously* and *irreducibly* toxic as well, i.e., heteronomizing, proletarianizing or disindividuating, no dialectial sublation and thus no perfect interiorization is possible. What is more, the autonomy of ‘the interior’ (‘the subject’, ‘the self’) *remains* *forever* dependent on exterior technical heteronomy: yes it is constituted by it, but only after its adoption as the formation of individual or social practices. For Stiegler, the ‘subjugating’ or ‘enslaving’ character of technology cannot be explained solely (and instrumentally) on the basis of the class struggle, i.e., from the social relations of production, as in Marxism. As a *pharmakon*, technology is *originally proletarianizing*, but for the very same reason it is also the (only) route towards deproletarianization.

In this regard, Verbeek is very right to point out that Stiegler provides ‘a fascinating new turn’ to the Marxist dialectic (although it is not a dialectic anymore as should be clear by now), in showing that ‘the struggle between humanity and technology is constitutive for the human being’, although the term ‘struggle’ does not seem to be very appropriate for what Stiegler theorizes as the human’s pharmacological relation with technology, in which the latter both con-stitutes and de-stitutes the former. I do not believe I’m regressing towards a traditional Marxist position in calling for the (re)appropriation of the digital *pharmaka* to reconquer our collective autonomy vis-à-vis capital, since this is not ‘a call to take up arms against the technologies of capitalism’ but a call to fight capitalism *with* the technologies ‘of capitalism’, but this attribution precisely becomes problematic of course from a pharmacological perspective.

Far from reinstating a separation of human beings and technologies, as Verbeek claims oddly enough, it is exactly a recognition of the need, the necessity, to reforge the *pharmakon*—which is *always* and by its very ‘*nature*’ a weapon, as Stiegler rightly insists time and again with reference to Deleuze—to regain social autonomy *on the basis of* the new technical heteronomy that the digital *pharmaka*, which are indeed not ‘mere vehicles of capitalism’, represent. True enough, whereas Stiegler only calls for a struggle against today’s capitalism’s proletarianizing and heteronomizing tendencies, I firmly side with the post-autonomists in calling for a technical fight against capitalism *as such*. In that sense, I advocate the challenge of ‘pre-given frameworks’ on the basis of ‘concrete technologies’ (‘from within’ if you like), as Verbeek suggests I should do, fully convinced that it is precisely technology in its very concreteness that represents the domain of the (empirical-accidental) transcendental because it is technologies, first of all mnemotechnologies like writing, printing and digital computing of course, that constitute and condition the possibilities of human thought, knowledge and critique in general, but to explain this would take too much space here (as would the plea for the necessity of a ‘transcendental (re)turn’ in philosophy of technology to make it properly philosophical again and rescue it from its myopic fascination with empirically describing the effects of the most recent techno-commodities on a consumber-subject that is not in any way problematized, and neither is their nature *as* *productive* of that subject).

That digital technologies ‘can also be the source of new forms of social agency and self-awareness’ is thus *exactly* what I claim, and also that ‘technologies are not opposed to politics’, precisely because ‘they are its very media’ (although I would prefer to say that they completely *re*-*constitute* and *re*-*condition* political action and even the political *as such* in our epoch): what could be more proof of ‘a realistic recognition of the technological condition of human existence’ than emphasizing that these technologies constitute a new *political organology* that has *yet* to be appropriated politically? It is precisely this new (digital) organological configuration that forms ‘the ecostructure in which we inevitably live our lives’, but as a fundamentally *pharmacological* milieu it is a *battlefield* as well as an *arsenal* that allows for a *struggle*—that is what politics *is*—against a destructive and utterly nihilistic capitalism that has for too long now claimed that it is ‘inevitable’ and without alternative (and about which postphenomenology remains totally silent).

It is true that there are no ‘close analyses of the technological things themselves’ in my article, but I do provide a description of the *general characteristics* of DNTs that make them ideally suited for the invention of an alternative, post-capitalist economy. Of course, empirical analysis always remains important in philosophy of technology, but analyzing technologies in complete *abstraction* from the politico-economic conjuncture in which they appear and operate, as postphenomenology usually does, is also pretty unrealistic in my view. Apart from that, and notwithstanding some notable exceptions (e.g. Van Den Eede 2012), one does not find that much analysis of (new) media, and neither a dialogue with media theory, in postphenomenology, which is strange for a philosophy that explicitly theorizes technological mediation.

Also, a philosophy of technology that has no eye for the libidinal aspects of technology (and technical artifactuality is precisely that which ‘mediates’ human affectivity and renders it different from the instinctual behavior of animals), and even questions the usefulness of the libidinal perspective, as Verbeeks seems to do, may lose sight of a significant ‘reality’-dimension of technology: that of the affective-phantasmatic. And a fortiori a ‘political theory of technology’ should take account of this, if it is true that political action and communication is about directing collective investment of desire. Both postphenomenology and critical theory of technology could profit in that regard from an engagement with psychoanalysis, of course based on an awareness of the thoroughly technologically conditioned ‘nature’ of the human psyche, its affects and its capacities.

## References

[CR1] Bantwal-Rao M, Jongerden J, Lemmens P, Ruivenkamp G (2015). Technological mediation and power: Postphenomenology, critical theory, and autonomist marxism. Philosophy and Technology.

[CR2] Berardi F (2009). The soul at work. From alienation to autonomy.

[CR3] Carr N (2010). The shallows. How the internet is changing the way we think, read and remember.

[CR4] Carr N (2015). The glass cage: Where automation is taking us.

[CR5] Crary J (2013). 24/7. Late capitalism and the ends of sleep.

[CR6] Dean J (2010). Blog theory. Feedback and capture in the circuits of desire.

[CR7] Dean J (2012). The communist horizon.

[CR8] Hardt M, Negri A (2012). Declaration.

[CR9] Keen A (2015). The internet is not the answer.

[CR10] McChesney R (2013). Digital disconnect. How capitalism is turning the internet against democracy.

[CR11] Pariser E (2011). The filter bubble: What the internet is hiding from you.

[CR12] Rouvroy, A., & Berns, T. (2013). Gouvernementalité algorithmique et Perspectives d’Émanicaption. Le disparate comme condition d’individuation par la relation? In *La Découverte|Réseaux* (Nr. 177, pp. 163–196).

[CR13] Stiegler B (2009). Acting out.

[CR14] Stiegler B (2015). States of shock stupidity and knowledge in the 21st century.

[CR15] Van Den Eede Y (2012). Amor technologiae.

